# Is There Any Evidence of Premature, Accentuated and Accelerated Aging Effects on Neurocognition in People Living with HIV? A Systematic Review

**DOI:** 10.1007/s10461-020-03053-3

**Published:** 2020-10-06

**Authors:** Htein Linn Aung, Maral Aghvinian, Hetta Gouse, Reuben N. Robbins, Bruce J. Brew, Limin Mao, Lucette A. Cysique

**Affiliations:** 1 Departments of Neurology and HIV Medicine, St Vincent’s Hospital and Peter Duncan Neurosciences Unit, St Vincent’s Centre for Applied Medical Research (AMR), Level 8, Lowy Packer Building, 405 Liverpool St, Darlinghurst, Sydney, NSW 2010 Australia; 2 Neuroscience Research Australia, Sydney, Australia; 3 Faculty of Medicine, UNSW, Sydney, Australia; 4 Department of Psychology, Fordham University, New York, USA; 5 Department of Psychiatry and Mental Health, University of Cape Town, Cape Town, South Africa; 6 HIV Center for Clinical and Behavioral Studies, New York State Psychiatric Institute, Department of Psychiatry, Columbia University Vagelos College of Physicians and Surgeons, New York, USA; 7 Faculty of Medicine, University of Notre Dame, Sydney, Australia; 8 Centre for Social Research in Health, UNSW, Sydney, Australia

**Keywords:** HIV/AIDS, Aging, HAND, Neuropsychology, Systematic review

## Abstract

Despite evidence of premature, accentuated and accelerated aging for some age-related conditions such as cardiovascular diseases in people living with HIV (PLHIV), the evidence for these abnormal patterns of aging on neurocognition remains unclear. Further, no systematic review has been dedicated to this issue. Using PRISMA guidelines, we searched standard databases (PubMed, EMBASE, CINAHL and PsycINFO). Articles were included if they analyzed and reported the effect of age on neurocognition among PLHIV as one of their major findings, if they were conducted in the combination anti-retroviral therapy era (after 1996) and published in a peer-reviewed journal in English. Quality appraisal was conducted using the Joanna Briggs Institute (JBI) appraisal tools. To systematically target the abnormal patterns of neurocognitive aging, we define *premature cognitive aging* as significant interaction effect of HIV status and age on cross-sectional neurocognitive test performance covering both the normal and abnormal performance range; *accentuated cognitive aging* as significant interaction effect of HIV status and age on cross-sectional neurocognitive impairment (NCI) rate, thus covering the abnormal performance range only; *accelerated cognitive aging* as significant interaction effect of HIV status and age on longitudinal neurocognitive test performance or incidence of NCI. Because these definitions require an age-comparable HIV-negative (HIV−) control group, when no controls were included, we determined the range of the age effect on neurocognitive test performance or NCI among PLHIV. A total of 37 studies originating from the US (26), UK (2), Italy (2), Poland (2), China (2), Japan (1), Australia (1), and Brazil (1) were included. Six studies were longitudinal and 14 included HIV- controls. The quality appraisal showed that 12/37 studies neither used an age-matched HIV- controls nor used demographically corrected cognitive scores. A meta-analysis was not possible because study methods and choice of neurocognitive measurement methods and outcomes were heterogeneous imposing a narrative synthesis. In studies with an HIV- control sample, premature neurocognitive aging was found in 45% of the cross-sectional analyses (9/20), while accelerated neurocognitive aging was found in 75% of the longitudinal analyses (3/4). There was no evidence for accentuated aging, but this was tested only in two studies. In studies without an HIV- control sample, the age effect was always present but wide (NCI OR = 1.18–4.8). While large sample size (> 500) was associated with abnormal patterns of cognitive aging, most of the studies were under powered. Other study characteristics such as longitudinal study design and higher proportion of older participants were also associated with the findings of abnormal cognitive aging. There is some support for premature and accelerated cognitive aging among PLHIV in the existing literature especially among large and longitudinal studies and those with higher proportion of older samples. Future HIV and cognitive aging studies need to harmonize neuropsychological measurement methods and outcomes and use a large sample from collaborative multi-sites to generate more robust evidences.

**Electronic supplementary material:**

The online version of this article (10.1007/s10461-020-03053-3) contains supplementary material, which is available to authorized users.

## Introduction

People living with HIV (PLHIV) are living longer than ever before with the widespread use of combination anti-retroviral therapy (cART) [1, 2]. The life expectancy of clinically stable PLHIV is approaching that of the non-HIV infected population [3], although this is hindered by those who have associated comorbidities [4]. As a result, the number and proportion of older PLHIV (over 50 years of age) are increasing [3, 5, 6]. UNAIDS [5] estimated that globally there were 5.8 million elderly PLHIV which account for 16% of the total PLHIV population, and it has increased to 7.9 million (21%) in 2019 [7].

Previous studies in HIV and aging have found that HIV may lead to premature, accentuated and even accelerated aging [8]. Age-related conditions such as cardiovascular diseases (CVD), frailty, chronic renal disease and stroke were observed at a higher rate and at an earlier age among PLHIV than age-matched people without HIV even when accounting for lifestyle factors [6, 9, 10]. Immunosenescence, driven by chronic inflammation, chronic immune activation, and microbial translocation processes in chronic HIV infection, has been suggested as the underlying pathological process [11, 12].

As PLHIV are aging, neurocognitive health is becoming as important as physical and mental health as age is the primary risk factor for dementia [8] and HIV itself is a risk factor for neurocognitive impairment (NCI) known as HIV-associated neurocognitive disorder (HAND). According to Frascati criteria [13], HAND is classified into three stages: Asymptomatic Neurocognitive Impairment (ANI), Mild Neurocognitive Disorder (MND), and HIV-associated dementia (HAD), and requires the use of demographically-corrected test scores as well as functional status and the exclusion of non-HIV causes of impairment. HAND, especially the ANI subtype still persists among PLHIV between 20–50% even in the cART era [14–18].

HIV may also accentuate and/or accelerate brain aging directly through chronic neuroinflammation [19, 20] and indirectly by promoting premature and accentuated systemic aging and comorbid conditions such as CVD and kidney disease that are themselves associated with NCI [21]. Therefore, it is important to understand whether HIV also leads to abnormal neurocognitive and brain aging as with other age-related conditions (i.e. whether HIV synergistically interacts with age to pose a greater risk for NCI or neurocognitive decline than the risk imposed by HIV or age itself). If this is true, PLHIV would systematically be at a much higher risk of dementia as they age, representing a major public health issue worldwide.

However, based on the literature, it is unclear whether HIV infection is associated with premature, accentuated and/or accelerated neurocognitive aging or whether there is a negative synergistic effect of HIV and age on neurocognition [22–24]. To the best of our knowledge there has been no systematic review on this topic. A systematic review may lead to a higher level of evidence, and importantly aid in identifying which factors may be associated with neurocognitive aging in PLHIV assisting in future research directions.

In this review, we defined abnormal patterns of neurocognitive aging as follows: *premature cognitive aging* represents significant interaction effect of HIV status and age on cross-sectional neurocognitive test performance covering both the normal and abnormal performance range (i.e., HIV and older age synergistically lead to significantly poorer neurocognitive performance compared to HIV or/and aging effect alone); *accentuated cognitive aging* represents significant interaction effect of HIV status and age on cross-sectional NCI rate, thus covering the abnormal performance range only (i.e., HIV and older age synergistically lead to much greater NCI rate compared to HIV or/and aging effect alone); *accelerated cognitive aging* represents significant interaction effect of HIV status and age on longitudinal neurocognitive test performance or incidence of NCI (i.e., HIV and older age synergistically lead to much steeper neurocognitive decline or significantly higher incidence of NCI compared to HIV or/and aging effect alone). Based on these definitions, it is possible that a cross-sectional study may only be able to detect premature aging and accentuated aging. A longitudinal study, though, may be able to detect not only premature and accentuated aging at baseline, but also accelerated aging at follow-up. However, these definitions necessitate the inclusion of an HIV-negative (HIV-) control group. Therefore, when an HIV- control group was not included, we were only able to determine the size of the aging effect within the HIV-positive (HIV +) participants, whether cross-sectionally or longitudinally, but not premature, accentuated or accelerated aging per se.

To achieve a comprehensive overview of the literature, we focused on the effect of aging on overall neurocognitive test performance or NCI rather than strictly following the HAND diagnosis criteria [13]. HAND is a diagnosis of exclusion that was conceptualized well before the aging effects of HIV were anticipated; and it is not possible to fully exclude non-HIV age-related conditions that may contribute or compound HIV-related NCI [24]. This strategy also ensures a more representative review because not all studies have correctly and comprehensively applied current standard diagnostic for HAND [25].

The overarching aim of this review was to synthesize and evaluate results derived from the existing literature in observational/interventional cross-sectional and longitudinal cohort studies conducted during the cART era in order to determine the extent of aging effects on neurocognition among PLHIV. More specifically, we first aimed at determining the magnitude of the age effect on the prevalence and incidence of NCI and overall neurocognitive test performance among PLHIV; and secondly, we aimed at determining the evidence for premature, accentuated and/or accelerated aging effects on NCI and overall neurocognitive test performance among PLHIV compared to HIV- controls.

## Methods

### Search Strategy and Selection Criteria

This review was carried out according to the Preferred Reporting Items for Systematic Reviews and Meta-Analyses (PRISMA) guideline [26]. A protocol for this review was registered on the PROSPERO website on 19/02/2019 (Registration ID—CRD42019123952). PubMed, EMBASE, CINAHL and PsycINFO databases were searched using the search terms presented in the Supplementary File 1 to find relevant literature, and this was conducted on 04/02/2019 (an example of the search is presented in the Supplementary File 1). Citation lists of the eligible articles from these databases were also manually searched for any additional relevant articles. A manual search was conducted again in Google Scholar on 20/12/2019 to find the articles published after the last database search.

The following inclusion/exclusion criteria were applied for the selection of studies. Studies were included if they analyzed and reported the effect of age on neurocognition among PLHIV as one of their major findings, if they were conducted in the cART era typically after 1996, if they included only adult participants (aged > 18 years), and if they included PLHIV of whom > 30% were on cART. Studies were excluded if they were not published in a peer review journal, if they were not written in English, and if the sample size was < 30.

Articles were initially screened from reading titles and abstracts by HLA. After this initial screening process, full-text articles of all the relevant articles were obtained. The full texts were then reviewed by HLA and independently by MA to assess eligibility criteria. A consensus discussion was conducted with LC when eligibility was uncertain. Final selection of the articles was made by matching the articles chosen between two reviewers.

Data were extracted by both HLA and MA using a Microsoft Excel format developed by HLA which covered the following areas: Study Characteristics, Study Method and Results (see the Supplementary File 2 for the detailed areas assessed). Data collected by two reviewers were compared; and any mismatched information was discussed again with LC to form a final consensus.

### Data Analysis

Critical appraisal tools from Joanna Briggs Institute (JBI) [27] for analytical cross-sectional studies and cohort studies were used to review the quality of the eligible articles. The JBI tools were adjusted to be more relevant to the topic of this review. Specifically, we first specified whether the study included a demographically comparable HIV- control group and/or used demographically corrected cognitive scores. Further, we adapted the rating classification across the JBI tools from “Yes, No, and Not Clear” to “Yes, Partly, No, and Not Applicable” to better rate the neuropsychology methodology of each study. The detailed definitions of all the items assessed are presented in the Supplementary File 3. The quality assessment was conducted independently by both HLA and MA. Any discrepancy on the quality ratings between the reviewers was discussed with LC to arrive at a final consensus. No study was dismissed a priori as the systematic review intended to provide a transparent snapshot of the quality of the cognitive aging literature in HIV at this moment in time.

A meta-analysis could not be conducted as initially intended because study designs, methods and choice of neuropsychological measurement methods and outcome variables were too heterogeneous among studies. We therefore used a narrative synthesis approach [28] to integrate the review findings. When synthesizing and comparing results across studies, we presented some figures with some quantitative outcomes, but they only represent descriptive aspects of studies. Outcomes from the studies were synthesized separating studies with and without an HIV- control group. From the studies with HIV- controls, the presence and magnitude of premature and accentuated (from cross-sectional studies) or accelerated aging effects (from longitudinal studies) were interpreted by evaluating the presence of an interaction effect between HIV and age on neurocognition. In studies without an HIV- control group, we were able to only identify whether there was a significant aging effect on the level of neurocognitive test performance or the prevalence and incidence of NCI/HAND within the HIV + participants. We also extracted and presented the range of effect size of aging effect on neurocognition among the studies without HIV- controls. However, studies which did not use demographically corrected scores were not included in the reporting of effect sizes. This was done to present the best interpretable data regarding the effect of chronological age in PLHIV versus normal aging.

## Results

**Fig. 1:**
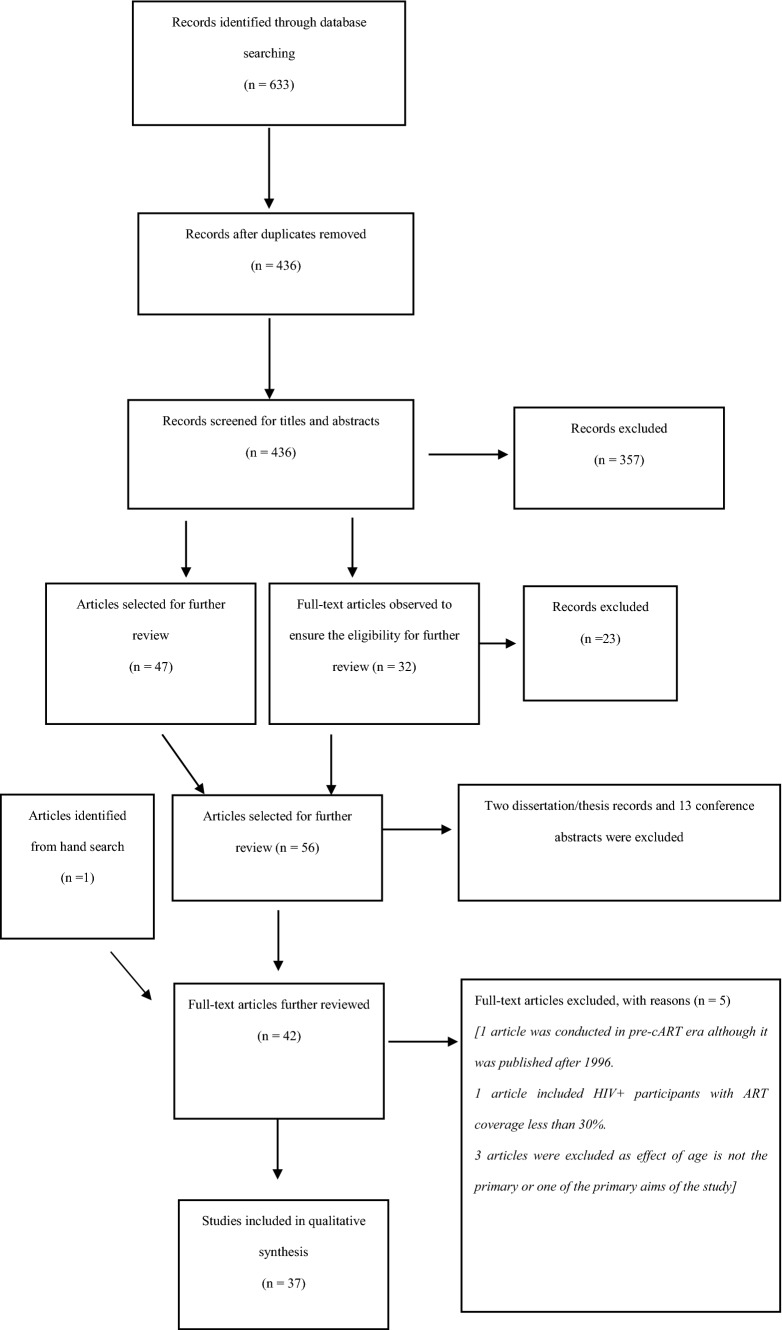
CONSORT Flow diagram for literature searching

The database search process returned a total of 436 articles. After screening, 37 articles (31 cross-sectional and 6 longitudinal) were selected (see the PRISMA Flow Chart in Fig. 1 for detailed screening process). Studies’ characteristics are presented in Table 1 for studies that included an HIV- control group and Table 2 for studies that did not include a control group, whereas the methods and outcomes of the studies are detailed in Table 3 (studies with an HIV- control group) and Table 4 (studies without a HIV- control group).

**Table 1:** Characteristics of studies that included a HIV- control group

Author	Country	Context	Sample Size	Age range	% of over 50	% of ART patients	% of plasma viral suppression or Mean/Median plasma viral load count	% of CSF viral suppression or Median CSF viral load count	% by disease stage (CDC Stage C/AIDS/WHO Stage 4	Mean or Median Nadir CD4 Count	Mean or Median CD4 Count	Mean or Median duration of HIV infection or serostatus (years)	Mean or median duration of ART (years)
Sheppard et al. (2017) [57]	US	From two cohort studies: HNRP at University of California San Diego (UCSD) and a study on healthy aging at UCSD and the VA San Diego Healthcare System	128 (40 HIV + and 88 HIV−)	50–75	100%	93%	85%	85.20%	57.50%	184	590.5	17.9	NR
Vance et al. (2011) [51]	US	Recruited from Birmingham, Alabama area through advertisements and word-of-mouth	201 (98 HIV + and 103 HIV−)	23–67	24%	NR	51%	NR	NR	NR	491	12.05	NR
Ludicello et al. (2012) [54]	US	From HNRP	257 (113 HIV + and 144 HIV−)	NR	44%	59%	Median Log10–3.18	NR	51%	210	425	8	NR
Goodkin et al. (2017) [43]	US	From MACS Study, a long-term cohort study with MSM with or without HIV	5086 (2278 HIV + and 2808 HIV−)	18–NR	8%	100%	41%	NR	5%	409	497.9	5.77	NR
Haynes et al. (2018) [29]	UK	From Brighton and Sussex University Hospitals, Kings College Hospital, Royal Free Hospital, St George’s Hospital and St Mary’s Hospital Stable HIV patients (asymptomatic, undetectable viral load, on ART at least 6 months, no comorbidities, no substance or alcohol abuse). All are Caucasian MSM	55 (30 HIV + and 25 HIV−)	NR	56%	100%	100%	NR	NR	175	741.7	14.54	10.57
Ding et al. (2017) [33]	China	From the Taizhou prefecture of Zhejiang province in China. Controlled subjects were from those receiving voluntary confidential counselling and testing or routine examination at the local center for disease prevention and control	690 (345 HIV + and 345 HIV−)	40–82	50%	87%	92.60%	NR	NR	> 200 = 44.3%	> 350 = 51.6%	3	2.2
Sacktor et al. (2010) [44]	US	From MACS study. All are MSM	1276 (477 HIV + and 799 controlled)	NR–65	51%	71%	Mean Log10–2.57	NR	NR	236	548.15	15.4	NR
Avci et al. (2016) [52]	US	From local HIV clinics and community in the San Diego	314 (189 HIV + and 125 HIV−)	NR	60%	88%	80%	NR	53%	222	583.4	12.95	NR
Seider et al. (2014) [45]	US	From the outpatient Immunology Centre of the Miriam Hospital and the Brown University Centre for AIDS Research	84 (54 HIV + and 30 HIV−)	40–74	27%	93%	78%	NR	NR	153	420	14.3	NR
Morgan et al. (2011) [59]	US	From a study funded by National Institutes of Mental Health	166 (126 HIV + and 40 HIV−)	NR	32%	83%	Median Log10–2	Median Log10–2	60%	163	517.93	13.47	NR
Scott et al. (2011) [53]	US	From community organizations and local HIV clinics	116 (44 HIV− and 72 HIV +)	NR–79	59%	72%	69%	83.00%	58%	152	554.67	13.6	8.88
Wilkie et al. (2003) [41]	US	Participants were recruited through community events, advertisements in local media, pamphlet distribution and through referral from local HIV/AIDS service/research centers, the University of Miami School of Medicine/Jackson Memorial Medical Centre and Miami Veterans Affairs Medical Centre	149 (63 HIV− and 86 HIV +)	20–NR	46%	90%	Mean Log10–2.36	NR	38%	NR	396.17	NR	NR
Sheppard et al. (2015) [42]	US	From a NIH funded study on aging and memory in HIV disease	146 (83 HIV + participants)	NR	61%	79%	75%	NR	55%	206.94	556.54	12.79	NR
Ciccarelli et al. (2012) [32]	Italy	From the Memory clinic, Catholic University of the Sacred Heart, Rome, Italy through outpatient clinic visits. Asymptomatic and free from opportunistic infections at the time of recruitment	192 (153 HIV + and 39 HIV−)	NR	25%	88.20%	77.90%	NR	26%	166	518	12	9
Gawron et al. (2018) [35]	Poland	From the Hospital of Infectious Diseases in Warsaw. HIV- controls were recruited from the local community Stable patients (on cART for at least 10 months and viral load < 60 copies per micro liter)	186 (91 HIV + and 95 HIV− participants)	25–NR	29%	100%	100%	NR	NR	272.9	597.3	5	5
Cysique et al. (2011) [39]	Australia	From St Vincent's Hospital in Sydney	145 (115 HIV + and 30 HIV−)	28–70	46%	100%	52%	NR	NR	87.2	326	NR	NR
Pluta et al. (2019) [36]	Poland	From the Harmonia 3 research project which had recruited participants from the Central Hospital for Infectious Diseases in Warsaw The participants were MSM who had been stabilized on ART for at least six months	115 (53 HIV + and 62 controls)	24–75		100%	100%	NR	NR	275.57	597.81	6.14	5.07
Towgood et al. (2012) [30]	UK	Participants were White/Caucasian MSM. Stable on ART (viral load < 50 copies/ml and CD4 > 200 cells/ml for at least six months)	82 (40 HIV + and 42 HIV−)	20–75	51%	100%	100%	NR	NR	173.05	665.98	10.55	6.93
Valcour et al. (2011) [55]	US	From Hawaii Aging with HIV (HAHC) Cohort Age, education, ethnicity and gender matched HIV- controlled participants were also recruited in the HAHC cohort	450 (204 HIV− and 246 HIV +)	NR	54%	51%		NR	NR	< 200 = 37%	< 200 = 40%	NR	NR
Vance, Fazeli and Gakumo (2013) [66]	US	From a university clinic in Birmingham, Alabama, metropolitan area	162 (84 HIV− and 78 HIV +)	20–74	44%	100%	Mean—14,671.85 copies/ml	NR	NR	276.38	471.3	12.93	NR
Kissel, Pukay-Martin and Bornstein (2005) [40]	US	From AIDS Clinical Trials Unit All were MSM and majority were Caucasians	241 (61 HIV− and 180 HIV +)	20–60	13%	NR	NR	NR	26%	NR	400.96	NR	NR
Kim et al. (2008) [50]	US	From eight public clinics run by a university medical center. HIV- participants were those receiving primary care services from two of these eight clinics	275 participants (91 HIV + and 184 HIV−)	18–NR	33%	NR	NR	NR	NR	NR	NR	NR	NR

*HIV* + HIV-positive. *HIV* − HIV-negative. *%* percentage. *CSF* cerebrospinal fluid. *ART* antiretroviral therapy. *CDC* centers for disease control and prevention. *AIDS* acquired immunodeficiency syndrome. *WHO* World Health Organization. *HNRP* HIV neurobehavioral research program. *MACS* multicenter AIDS cohort study. *NIH* National Institute of Health. *MSM* men who have sex with men. *NR* not reported

**Table 2:** Characteristics of studies that did not include a HIV—control group

Author	Country	Context	Sample size	Age range	% of over 50	% of ART patients	% of plasma viral suppression or mean/median plasma viral load count	% of CSF viral suppression or median CSF viral load count	% by disease stage (CDC Stage C/AIDS/WHO stage 4	Mean or median Nadir CD4 Count	Mean or median CD4 count	Mean or median duration of HIV infection or serostatus (years)	Mean or median duration of ART (years)
Kupprat et al. (2015) [46]	US	From global opportunities for leadership development project	199	50–69	100%	94%	80.50%	NR	NR	NR	482.51	17.76	NR
Pinheiro et al. (2016) [38]	Brazil	From a specialized center for HIV/AIDS in the city of Pelotas, South Brazil	392	50–82	29%	89%	65%	NR	NR	< 200 = 38%	< 200 = 4%	7.55	5.62
Kinai et al. (2017) [37]	Japan	From 17 facilities across Japan	728	20–NR	32%	97%	86.50%	NR	32%	163.4	549.7	7.62	6.8
Cherner et al. (2004) [56]	US	From studies conducted by UCSD on the neurobehavioral consequences of HIV	119	NR–67	56%	53%	41%	50%	73%	NR	274.13	9.67	NR
Cohen et al. (2019) [47]	US	From Brown University Centre for AIDS Research	104	NR	45%	97%	75%	NR	49%	221.5	567.6	17.5	NR
Valcour et al. (2004) [62]	US	From all major islands in Hawaii	198	20–NR	52%	72%	44%	NR	NR	223.35	452.37	9.84	NR
Larussa et al. (2006) [31]	Italy	Secondary data from the Italian Registry Investigative Neuro AIDS (IRINA) which recruits HIV patients diagnosed with a neurological disease from 45 infectious disease centers in Italy	195	20–NR	20%	45%	Mean Log10—3.74	NR	29%	NR	143.63	NR	3
Coban et al. (2017) [49]	US	From the AIDS Clinical Trials Group	3313	NR		100%	91%	NR	NR	> 350 = 21%	> 350 = 79%	NR	3.5
Sacktor et al. (2007) [63]	US	Secondary data from Hawaii Aging with HIV Cohort Study	254	20–NR	52%	79%	46%	NR	NR	223.34	454.28	9.8	NR
Tan et al. (2013) [64]	US	From the Johns Hopkins University NIMH Centre Clinical Outcome Cohort	106	NR	30%	100%	Mean Log10—1.22	Mean Log10–0.89	NR	90.05	374.84	13.15	NR
Sandkovsky et al. (2013) [48]	US	Based at the University of Nebraska Medical Centre	41	20–70	49%	100%	83%	NR	NR	239.98	682.49	9.95	7.47
Van Dyk et al. (2015) [65]	US	NR	42	30–75	59.52%	93%	77%	NR	NR	NR	NR	16.67	NR
Panos et al. (2013) [58]	US	From the National NeuroAIDS Tissue Consortium	259	NR	22.00%	85%	NR	NR	NR	NR	219.35	11.26	NR
Foley et al. (2010) [60]	US	Recruited from the community agencies and medical centers in Los Angeles	98	NR	27.55%	NR	Log10–8.24	NR	58%	Log10–4.64	Log10–5.77	NR	NR
Xiao et al. (2019) [34]	China	From two main HIV clinics in Hunan province Inclusion criteria: age ≥ 60 and currently taking ART	250	60–80	100%	100%	NR	NR	NR	NR	NR	NR	NR

*HIV* + HIV-positive. *HIV* − HIV-negative. *%* percentage. *CSF* cerebrospinal fluid. *ART* antiretroviral therapy. *CDC* Centers for Disease Control and Prevention. *AIDS* acquired immunodeficiency syndrome. *WHO* World Health Organization. *UCSD* University of San Diego. *NIMH* National Institute of Mental Health. *NR* not reported

**Table 3:** Methods and findings among studies that included HIV − controls

Author	Study design	Main exposure variable	Type of neuropsychological (NP) tests	No of neurocognitive domains tested	Outcome measure (domain score/test score/NCI)	Main finding (premature/accentuated/accelerated)	Summary of findings
Premature neurocognitive aging (yes/no)							
Sheppard et al. (2017) [57]	Cross-sectional	Age groups (50–65 VS ≥ 65) and HIV serostatus	Standard	4	Domain (z score)	Premature	HIV + group (50–65 years) performed more poorly than HIV- group [50–64] but not differently from HIV- group (over 65 years) in Digit Span test and initial recall of the Supraspan Word List test which cover auditory verbal attention domain
Vance et al. (2011) [51]	Cross-sectional	Age groups (< 50 VS > 50) and HIV serostatus	Standard	9	Test (raw score)	Premature	Age and HIV interaction effect was detected in Trial Making Test A and Complex Reaction Time. Poorer performance was reported by older HIV + participants
Iudicello et al. (2012) [54]	Cross-sectional	Age groups (< 40 VS > 50) and HIV serostatus	Standard	7 (primary—verbal fluency)	Domain (T score) and Frascati	Premature	J-T statistic showed the additive effect of HIV and age on the overall verbal fluency performance in both category and letter fluency. Older HIV + group performed the worst among the four groups. Similar effect was also observed in the switching component of the verbal fluency performance although this effect was not found for the clustering component
Goodkin et al. (2017) [43]	Longitudinal (six monthly follow-up data collection)	Age (continuous for regression and group (< 50 VS ≥ 50) for comparative analysis) and HIV serostatus	Standard	5	Domain (T score)	Premature	The final regression model including time since seroconversion and other significant controlled variables showed that the age and HIV interaction had a negative effect on motor function and episodic memory (HIV- VS AIDS and symptomatic stage) and a positive effect on working memory
Avci et al. (2016) [52]	Cross-sectional	Age (< 40 VS > 50) and HIV serostatus	Standard	6 (primary—prospective memory)	Domain (T score)	Premature	Logistic regression showed that the HIV and age interaction was significantly related to the naturalistic prospective memory (PM). In terms of self-perceived PM task, significant effects of HIV, depression/anxiety, and the interaction between HIV and age were identified
Morgan et al. (2011) [59]	Cross-sectional	Age (continuous for regression and group (< 50 VS ≥ 50) for comparative analysis) and HIV serostatus	Standard	9	Deviation (z score)	Premature	Regression analysis showed the significant interaction effect of age and HIV on the intra-individual variability. Follow-up analysis showed that older HIV + participants had higher dispersion than older seronegative group
Wilkie et al. (2003) [41]	Cross-sectional	Age (20–39 VS > 50 years) and HIV serostatus	Standard	4	Domain (z score)	Premature	In the information processing speed domain, effects of age and its interaction with HIV were observed. When contrasting group means, older HIV + participants and younger HIV + participants performed slower than their counterpart
Scott et al. (2011) [53]	Cross-sectional	Age groups (< 40 VS > 50) and HIV serostatus	Standard	4 (primary—cortical hypothesis)	Domain (raw score)	Premature	Interaction effect of age and HIV was detected in the recognition discriminability and post-hoc analysis showed that the difference was mainly contributed by the superior performance of younger HIV- participants
Ding et al. (2017) [33]	Cross-sectional	Age groups (40–49 VS 50–59 VS ≥ 60) and HIV serostatus	IHDS and MMSE	2	Domain and composite (raw score)	Premature	The percentage of NCI was the highest among the oldest HIV + participants. ANCOVA analysis reported that the interaction effect between HIV and Age was detected in motor speed, orientation, registration, recall and marginally in IHDS and MMSE composite scores. Post-hoc analyses indicated that this difference mainly came from the inferior performance of the oldest HIV + group (all four domains), and partially from the greater performance among the youngest HIV − (in recall domain)
Pluta et al. (2019) [36]	Cross-sectional	Age as a continuous variable and HIV serostatus	Standard	7	Domain (z score)	No premature age effect	Multiple regression analysis showed that HIV and age were significantly associated with the complex attention score, but their interaction effect was not observed. Age was also identified as a significant predictor of the grey matter volume
Towgood et al. (2012) [30]	cross-sectional	Age (20–40 VS 50–75) and HIV serostatus	Standard	7	Test (raw score)	No premature age effect	Older participants performed worse in the Rey Auditory Verbal Learning Test (RAVLT) total words recalled, RAVLT immediate recall, RAVLT delayed memory recall and Word-Pairs false acceptance rate. A significant difference was found between HIV + and HIV- participants only in visual reproduction task. However, there was no interaction effect of HIV and age on any of the NP tests
Valcour et al. (2011) [55]	Cross-sectional (baseline figure of the cohort)	Age (< 40 VS > 50) and HIV serostatus	Standard	4	Test (z score)	No premature age effect	HIV + participants performed worse in all the summary scores compared to HIV − participants. Linear regression analysis showed that older HIV + participants with CD4 < 200 performed worse compared to HIV − young participants in Rey Complex Figure Delayed Recall and Digit Span Backwards while older HIV + with nadir CD4 > 200 performed worse in Timed Gait than HIV- participants. However, no interaction effect of HIV and age was reported
Vance, Fazeli and Gakumo (2013) [66]	Cross-sectional	Age groups (< 50 VS ≥ 50), HIV serostatus, and duration of serostatus	Standard	4	Test (raw score)	No premature age effect	Significant effects of age were observed on all the NP tests except Wisconsin Card Sorting Test. There were significant group differences between HIV + and HIV- participants in Useful Field of View, Complex Reaction Time Test, and Letter Comparison and Pattern Comparison test performances. But the interaction effect of age and HIV was not shown in any of the NP tests measured
Kim et al. (2008) [50]	Cross-sectional (real time research methodology was pursued in this study.)	Age groups (< 50 years VS ≥ 50 years) and HIV serostatus	Executive Clock Drawing Task	1 (primary—executive function)	Test (raw score)	No premature age effect	Younger participants had significantly higher scores in both Clock Drawing tests. Regression analysis also showed that older age was associated with poorer performance in both tests. However, no interaction effect was observed
Gawron et al. (2018) [35]	Cross-sectional	Age as continuous variable and HIV serostatus	Standard	6	Test (raw score)	No premature age effect	Both HIV and age independently contributed to the lower performance in NP test results although their interaction effect was not observed
Cysique et al. (2011) [39]	Cross-sectional	Age as continuous variable and HIV serostatus	Standard	6	Global impairment score and Frascati	No premature age effect	Both age and HIV status were independently associated in the expected direction to the global impairment score. However, their interaction effect was not clear. It was significant in the expected direction when age was included as a continuous variable and quadratic, and HAD cases were excluded
Accentuated neurocognitive aging (Yes/No)							
Ciccarelli et al. (2012) [32]	Cross-sectional	Age (< 60 VS ≥ 60) and HIV serostatus	Standard and MMSE	5	NCI (a pathologic stage was determined if a score is below the Italian normative cut-off (the lower margin of the tolerance interval of 95% for a 95% confidence level)) and Test (raw score)	No accentuated and no premature age effects	Both HIV and age showed an effect on both the number of pathological tests and individual test raw scores. However, no interaction effect of age and HIV was reported
Kissel, Pukay-Martin and Bornstein (2005) [40]	Cross-sectional	Age groups (20–35 VS 45–60), HIV serostatus and HIV staging (Stage A, B, and C)	Standard	7	NCI (a summary performance score was imputed based on the number of tests under one SD below the mean of the control group) and Test (raw score)	No accentuated and no premature age effects	Separate effects of HIV and age were observed in the summary performance score. However, their interaction effect was not found. Independent effects of HIV and age were also seen in individual neuropsychological test measures but no interaction effect was reported
Accelerated neurocognitive aging (Yes/No)							
Haynes et al. (2018) [29]	Longitudinal (an average follow-up period of 4.2 years)	Age groups (< 50 years VS ≥ 50 years) and HIV serostatus	Standard	5	Domain (z score)	Accelerated	HIV and age interaction effect was detected on the change in global neurocognitive score. Both older and younger HIV + participants performed worse than their HIV- counterparts. The worst performance was observed among the older HIV + group
Sacktor et al. (2010) [44]	Longitudinal (5 years and six-monthly visits)	HIV serostatus and Age groups (< 40 VS 40–50 VS ≥ 50)	Standard	2 (primary—psychomotor speed)	Test (raw score)	Accelerated but no premature age effect	Interaction effect of HIV, age and duration of follow-up was detected in the Trial Making Test B. Neurocognitive decline in this test was significantly higher in the older group compared to the younger group among HIV + participants while similar difference was not reported for the controlled group
Seider et al. (2014) [45]	Longitudinal (one-year follow-up)	Age groups (40–54 VS 55 +) and HIV serostatus	Standard	2 (primary—memory deficit)	Test (T score)	Accelerated but no premature age effect	The effects of age and its interaction with HIV status were significant on the change in Hopkins Verbal Learning Test-Revised (verbal memory) test. Only the older HIV + group declined in verbal learning and memory performance
Sheppard et al. (2015) [42]	Longitudinal (one-year follow-up)	Age groups (< 40 VS > 50 years) and HIV serostatus	Standard	6	NCI (sub-syndromic NCI was rated if a patient achieves a global clinical rating score ≥ 5 without any functional impairment. Syndromic NCI was defined if a patient scores ≥ 5 in the global clinical rating and if the patient meets two out of the 5 dependency/functional impairment criteria. The global clinical rating score was calculated based on test scores from the six neurocognitive domains which ranged 1 (above average, T score ≥ 55) to 9 (severely impaired, T Score < 20))	No accelerated age effect	Regression analysis revealed that only HIV status and neither age nor its interaction with HIV had a significant effect on the incidence of NCI. HIV + participants were 4.84 times more likely to develop NCI over the one-year follow-up period

*HIV* + HIV-positive. *HIV* − HIV-negative. *NCI* neurocognitive impairment. *IHDS* International HIV Dementia Scale. *MMSE* mini-mental state examination. *AIDS* acquired immunodeficiency syndrome. *HAD* HIV associated dementia

**Table 4:** Methods and findings among studies that did not include an HIV − sample

Author	Study design	Main exposure variable	Types of NP tests	No of neurocognitive domains tested	Outcome measure (domain score/test score/NCI)	Summary of findings
Kupprat et al. (2015) [46]	Cross-sectional	Age groups (50–54 VS 55–59 VS 60–64 VS 65–69) and Education levels (high school or less and more than high school education)	Standard and MMSE	4	Test (raw score)	Significant differences by age were detected for the Trial Making Test B and Similarities test
Pinheiro et al. (2016) [38]	Cross-sectional	Age groups (< 50 years and ≥ 50 years)	Standard, IHDS and montreal cognitive assessment	4	NCI (if a patient scored < 10 in IHDS and if the score in three out of the other five tests fell in the upper quartile)	Prevalence of NCI was significantly higher in over 50 years age group (53.5% VS 63.7% according to IHDS cut off and 29.1% VS 53.2% according to the study definition). Multivariate analysis showed that over 50 years of age was associated with 2.28 times higher risk of having NCI
Kinai et al. (2017) [37]	Cross-sectional	Age groups (< 50 years and ≥ 50 years) and duration of serostatus	Standard	8	Frascati	Patients diagnosed with asymptomatic NCI were significantly older (53.5% among over 50 VS 12.1% overall). According to the regression analysis, being over 50 had a 22% higher chance of symptomatic neurocognitive impairment (MND and HAD). Since the age of 50, the prevalence of symptomatic NCI abruptly increased, and this difference was statistically significant
Cherner et al. (2004) [56]	Cross-sectional	Age (< 35 VS > 50)	Standard	7	NCI (clinical ratings were assigned on a nine-point scale in which: 1, above-average functioning; 2, average; 3, below average; 4, borderline; 5, definite mild impairment; 6, mild-moderate impairment; 7, moderate impairment; 8, moderate-severe impairment; and 9, severe impairment. Global impairment was defined if two or more domains are scored in the impaired range)	Although more older participants met impaired criteria than younger participants in overall performance or individual domains, the differences between young and old age groups were not statistically significant. Logistic regression showed that age (X2 = 3.84) and CSF viral load were independently and interactively associated with global NP impairment
Cohen et al. (2019) [47]	Cross-sectional	Age groups (< 50 VS ≥ 50), heavy alcohol use or note, and lifetime alcohol use disorder or not	Standard	6	Domain (T score)	In Hierarchical regression analysis with HIV biomarkers, age as a continuous variable was negatively associated with overall neurocognitive test performance (Beta = − 0.25), speed of processing (Beta = − 0.25), attention/executive function (Beta = − 0.25) and memory (Beta = − 0.30)
Valcour et al. (2004) [62]	Cross-sectional	Age groups (20–39 VS > 50)	Standard	4	AAN	Prevalence of both HAD and mild cognitive motor disorder (MCMD) were higher among the older group. The older group had 3.26 times higher risk of being diagnosed with HAD after adjusting for other variables
Larussa et al. (2006) [31]	Cross-sectional for baseline analysis and cohort for survival analysis	Age groups 20–39 VS 40–49 VS ≥ 50)	Standard	NR	AAN	Prevalence of HAD was higher in the oldest group compared to the younger groups. This difference was mainly contributed by the deficit among the ART naive group (7.2% VS 15.3% VS 27.3%). The odds of having HAD and MCMD among those aged over 50 years compared to those aged between 20–39 years were 4.89 and 1.81 respectively
Coban et al. (2017) [49]	Cross-sectional	Age as continuous (every 10-year increase)	Standard	3	NCI (the presence of deficit was defined as having a score of < -2 SD in one test or < − 1 SD in two tests)	Being a decade older was related to 1.18 times higher risk of having NCI even 2 years after initiation of ART. The probability of having NCI increased significantly in the fifth decade of life (41–50 years of age)
Sacktor et al. (2007) [63]	Cross-sectional	Age groups (< 50 years and ≥ 50 years)	Standard	3	Domain (z score) and Frascati	The older group performed worse in all three neurocognitive domains assessed. But both younger and older groups did not show impairment in any of the tests in the motor speed domain
Tan et al. (2013) [64]	Cross-sectional using the NP test results from the patients' last visit	Age groups (< 50 years and ≥ 50 years)	Standard	7 (primary—memory)	NCI (memory deficit was defined as z-scores < − 1.5 for the verbal and non-verbal memory domains in either one or more of the following tests: immediate recall, recognition, delayed recall or Rey Complex Figure (delayed))	About 34% of younger participants and 50% of older subjects had memory impairment. The mean Z scores of all the memory tests were lower in older subjects in relation to younger participants. Older age was associated with a 4.8-fold higher odds of memory deficits. Every 1-year increase in age was associated with a 1.11-fold higher odds of memory deficit
Sandkovsky et al. (2013) [48]	Cross-sectional	Age groups (20–40 VS > 50)	Standard	5	Domain (z score)	There was a significance difference between the younger and older age groups in neurocognitive test performance in the memory domain
Van Dyk et al. (2015) [65]	Cross-sectional	Age groups (< 50 VS ≥ 50) and physical health	Standard	3 (primary—processing speed)	Test (raw score)	Older participants took significantly longer reaction time than young persons in all interstimulus intervals. The independent effect of age was detected in all three levels of Simple Reaction Time tests (Beta = 0.39, 0.43 and 0.43), Grooved Pegboard test (Beta = 0.44), and Letter Fluency Test (Beta = − 0.36) showing older age was associated with longer duration
Panos et al. (2013) [58]	Cross-sectional	Age (continuous for regression and (< 50 VS ≥ 50 for group differences) and APOE	Standard	5	Domain (z score) and Frascati	There was no significant difference of HAND prevalence between e4 positive and negative groups in the younger age group but in the older age group. Independent effect of age was detected in executive function (Beta = − 0.02), working memory (Beta = − 0.02) and information processing speed domains (Beta = − 0.03)
Foley et al. (2010) [60]	Cross-sectional	Cardiovascular risks (diabetes, hypertension, myocardial infarction, or congestive heart failure) and Age group (< 50 VS ≥ 50) for ANOVA and as a continuous variable for other analyses	Standard	6	Domain (T score)	Older age predicted poorer performance in the verbal fluency domain (Beta = − 0.633). The interaction effect between age and CVD was also reported in the verbal fluency domain
Xiao et al. (2019) (34)	Cross-sectional	Age as continuous (every 10-year increase)	Montreal cognitive assessment	1	Composite (raw score)	The total score decreased with age in ANOVA test and the percentage of impaired (< 26 for those with above high school and < 25 for those with lower than high school) increased with age on Fisher exact test. Hierarchical regression analysis showed that older age is negatively associated with the global neurocognitive test performance score (Beta = − 1.295)

*HIV* + HIV-positive. *HIV* − HIV-negative. *NCI* neurocognitive impairment. *IHDS* International HIV Dementia Scale. *MMSE* Mini-Mental State Examination. *AAN* American Academy of Neurology criteria. *MND* mild neurocognitive disorder. *HAD* HIV associated dementia. *CVD* cardiovascular disease

### Study Characteristics

#### Locations and age of studies

The majority of the studies were conducted in the US while two studies originated from the UK [29, 30], Italy [31, 32], China [33, 34], and Poland [35, 36], and one was carried out in Japan [37], Brazil [38], and Australia [39]. No study was conducted in low-income countries. Over two thirds of the studies (28 studies) were published in the last decade (2011–2019). The oldest record included was published in 2003 [40, 41].

#### Study design

In terms of the study design, only 6 out of 37 studies were designed longitudinally [29, 31, 42–45]. None of the 37 studies were interventional. The follow-up periods among these six longitudinal studies were one-year follow-up in three studies, and 5 years, 4.2 years (average) and 4.7 years (average) respectively in the other three studies respectively.

Age was the only primary predictor in all studies, but in eight studies, age was investigated as a co-primary exposure along with a HIV-related variable such as duration of HIV infection [37] or a non-HIV related factor such as education [46] and alcohol use [47]. Neurocognition was also the sole primary outcome of interest in all but five studies [29, 30, 35, 36, 48], which also identified the effect of age on changes in neuroimaging.

#### Sample size

The majority of the studies included samples lower than 300 (29/37) and the median sample size across the studies was 192 (IQR: 116–267). Five studies [33, 37, 43, 44, 49] recruited more than 500 participants. Two multi-site studies included very large samples: one with 3313 participants [49] and another one with 5086 samples [43].

#### Inclusion of HIV- controls

Fifteen studies did not include HIV- controls. In studies that included a HIV- control group, the proportion of HIV- participants among total samples varied from 20–69% (≥ 50% were controls in 48% of studies). Compared to HIV- controls, HIV + participants were younger in four studies [43–45, 50] and older in two studies [29, 51]. There were also differences in other demographic factors such as education and ethnicity between HIV + and HIV- participants in 11 studies [40–45, 50–53], and they controlled these factors in the analyses.

#### Samples ascertainment, gender, and ethnicity

In 17 of 36 studies, participants were recruited from existing research projects, such as the HIV Neurobehavioral Research Program (HNRP), Hawaii Aging Cohort Study and Multicenter AIDS Cohort Study (MACS). In the remaining studies, participants were recruited from HIV treatment clinics or the community. Twenty eight studies excluded participants with a major confounding condition for HAND as delineated using in the Frascati Criteria [13] such as major neurological and psychiatric disorders, current substance abuse and history of head trauma with loss of consciousness more than 30 min. Six studies [29, 30, 36, 40, 43, 44] included men who have sex with men (MSM) participants only, and three [29, 30, 40] of these recruited only Caucasian participants. Two studies [35, 46] included only male participants. Two British studies [29, 30] and two Polish studies [35, 36] enrolled solely virally suppressed HIV + participants (plasma HIVRNA < 50cp/mL). One study [31] included only formally diagnosed HAND cases and compared the distribution of young and old age groups among mild and severe cases of HAND.

#### Samples’ HIV disease characteristics

Major disease markers such as duration since HIV diagnosis, nadir/current CD4 + T cell count, duration of ART and viral control level were also presented across studies, although there was heterogeneity in how the information was presented and how much detail was provided. In one quarter of the studies, all HIV + participants were on ART. The percentage of patients on ART was lower than 70% in four of the studies [31, 54–56], all published before 2013. In 13 studies, the proportion of HIV + participants on ART was compared between the younger and older groups. A higher proportion of older HIV + participants was reported to be on ART across studies.

Standard HIV disease staging was reported in 15 studies. The proportion of WHO Stage 4 or CDC Stage C varied between 5–73% among studies; and in eight studies more than 50% of HIV + participants had been diagnosed with WHO Stage 4 or CDC Stage C in eight studies.

Nadir CD4 + T cell count was reported in 27 studies, while 34 studies gave the current CD4 + T cell count among their HIV + participants. The mean or median nadir CD4 + T cell count was lower than 200 in 10 studies, and the current CD4 + T cell was higher than 500 among participants in 14 studies. The current CD4 + T cell count was differentiated between the younger and older groups in 23 studies, and the younger group had a higher level in 13 of the studies. Nineteen studies compared the nadir CD4 + T cell count level between the age groups as well, and a higher proportion (15/19) of studies showed that the older participants had lower nadir CD4 + T cell counts.

Plasma viral load information was reported in 33 of 37 studies. The percentage with plasma viral suppression was reported in 26 of 36 studies. In 17 of 26 studies, ≥ 75% of participants had achieved plasma viral suppression. When the percentage of participants with plasma viral suppression was compared between young and old groups, it was higher among the older groups in 10 of 13 studies which assessed this question. Cerebrospinal fluid (CSF) viral load was reported in only five studies. Among them, the percentage of participants with CSF viral control was reported in three studies (50% in one study [56] and > 80% in another two studies [53, 57]).

Twenty-eight studies reported the mean or median duration of HIV infection or serostatus (range 3–18 years). In 17 of 28 studies, HIV duration was greater than 10 years. Mean or median duration of ART was stated only in 12 studies and varied from 2—11 years.

#### Reporting of comorbidities

Other known contributing factors to neuropsychological performance were collected across studies, but again with some heterogeneity in the level of detail. Mood status (anxiety or/and depression) was only assessed in 26 of 37 studies. Alcohol or substance use was reported in 18 of 37 studies. Co-infection with HCV or HBV was also reported in 14 of 37 studies. Information on age-related comorbidities such as hypertension (10/37 studies), diabetes (9/36) and CVD events (4/37) were scantly reported.

Across studies, findings indicated that HIV + participants compared to the HIV- controls tended to have higher prevalence of anxiety, depression and substance abuse. Further, across studies, older participants were more likely to have age-related comorbidities such as hypertension and were less likely to be a current substance user.

#### How was age defined?

The age of the youngest person included across the studies was 18 years old and the oldest person included was 82 years old. All but three studies investigated the effect of age across the adult life span. The three studies examined the effects of age only among participants over 50 years of age [34, 46, 57]. Age was categorized in 27 studies for major analysis and nine studies analyzed age as a continuous variable [34–36, 39, 43, 49, 58–60]. In most instances, age was categorized into two groups while four studies [31, 33, 44, 46] divided age into more than two groups. The lower cut-off point for the older group was 50 years of age in all studies, except three studies [32, 40, 45], where the lower bound was 45, 55 and 60 years respectively. Further, the upper bound of the younger age group was not consistent across all the studies. Some studies used < 50, whereas some studies adopted 35 or 40 years as upper limit of the younger age group. Apart from the three studies that only included participants over 50 years of age, the average proportion of cases over 50 years of age across the rest of the studies was 40% but with a wide range: 8–61%.

#### How was the cognition measured and defined?

All of the studies used comprehensive neuropsychological tests to measure neurocognitive performance, and three studies used screening tests like the International HIV Dementia Scale (IHDS) and Montreal Cognitive Assessment (MoCA) [33, 34, 38]. Only six [32, 35, 42, 50, 54, 59] out of 22 studies with HIV- controls and nine [31, 37, 48, 49, 56, 61–64] out of 15 studies without a control group standardized cognitive test scores with normative data. Twenty-six out of 37 studies explored the effect of age on a continuous measure of overall or domain specific neurocognitive performance, while 10 studies assessed the effect of age on study-defined NCI criterion. One study examined the age influence on neuropsychological test scores dispersion [59]. Among the studies that had NCI as a major outcome, two studies [31, 62] used the American Academy of Neurology (AAN) criteria [13] and three studies [37, 42, 56] used the Frascati criteria to define NCI. The remaining five studies adopted customized impairment criteria [32, 38, 40, 49, 64]. In one study [38] that used the IHDS and MOCA, the cut-off for NCI was < 10 for IHDS and ranked in the first quartile for MOCA. Among studies which measured the age effect on continuous neurocognitive performance, 16 studies analyzed on domain scores while 10 studies evaluated individual test scores.

#### Global cognition versus cognitive domains

The majority of studies (75% or 28/37) focused on global cognition or multiple cognitive domains. Nine studies selectively assessed age effects on specific cognitive domains, for example, verbal fluency [54] and memory [45, 52, 64], or a group of cognitive functions that underlie a neural pathway [53]. Among studies that focused on overall neurocognitive performance, 2 out of 3 assessed five or more cognitive domains. All the domains covered in the studies are aligned within the neurocognitive domains recommended to be evaluated in the Frascati criteria [13]: “Attention-information processing, language, abstraction-executive, complex perceptual motor skills, memory, including learning and recall, and simple motor skills or sensory perceptual abilities”.

#### HAND prevalence

Only eight out of 36 studies (22%) reported HAND prevalence [35–37, 39, 53, 58, 62, 64]. Of these, one study reported the prevalence only among older HIV + participants [54]. Seven studies used the Frascati criteria while one study used AAN criteria [62]. The prevalence of HAND varied highly among the reported studies ranging from 15 to 89%. The lowest prevalence was observed in a study [35] that included only high functioning HIV + participants. The highest prevalence rate of HAND was found in a study where a large proportion (81%) of HIV + participants had substance use disorders [64]. Four out of eight studies [36, 37, 39, 53] classified HAND clinical sub-categories, with ANI being the most prevalent (range: 53%-74%) in three studies, while MND was the predominant classification in one study (55%) [39]. Four of the studies compared HAND prevalence between age groups [37, 58, 62, 64]. HAND was found to be higher among younger participants in one study possibly because of higher cognitive reserve among older participants [58].

#### Statistical methods to assess the age effect

Statistical methods used to identify the effect of age on neurocognition among HIV + participants (versus HIV- when appropriate) varied across studies. Chi-square test or Fisher’s exact test, t-test or Wilcoxon test and Analysis of Variance (ANOVA) test were used to compare group differences in exploratory analyses depending on the type of predictor variables. Two-way ANOVA or Analysis of Covariance (ANCOVA), linear regression, logistic regression, generalized linear model, and linear mixed model were used for the main analysis to identify the interaction effect of HIV and age on neurocognition among studies which included HIV- controls. In studies without HIV- participants, hierarchical regression, linear regression, logistic regression, generalized estimating equation model, and cox proportional hazard model were adopted as a primary analysis method to identify the effect of age on cognition among PLHIV. The type of neuropsychological scores (i.e. raw scores, T-scores, z-scores) used for analysis also differed across studies. Z-score was the most common type of score (14/37 studies) followed by the raw score (13/37 studies), and T-score (10/37 studies). Attempts were made in all the studies except in one [50] to control for possible confounding factors such as non-age demographic factors, HIV biomarkers (e.g. nadir CD4), comorbidities, substance use and depression, and factors which are significantly different between HIV + and HIV- participants.

### Risk of bias within studies

Detailed appraisal outcomes for each question in the questionnaires are presented in Tables 5 and 6 (findings for individual studies are presented in the Supplementary File 3). Among cross-sectional studies, 22/31 studies meet the criteria for “Yes” or “Partly” across all the appraisal questions. Eight studies [34, 38, 46, 50, 51, 58, 60, 65] neither included age-matched HIV- controls nor used demographically corrected cognitive test scores. One study [41] did not give clear inclusion criteria. Confounding factors were not identified and controlled for in another study [50]. Among six longitudinal studies, only one study [31] met the criteria for “Yes” or “Partly” in all the questionnaires. One study [42] did not report how attrition was handled. Four other studies [29, 43–45] did not include age-matched HIV- controls or did not use demographically corrected cognitive test scores.

**Table 5:** Rating on quality appraisal among cross-sectional studies with the JBI tools

	Yes (%)	Partly (%)	No (%)	NA (%)
Were the criteria for inclusion in the sample clearly defined?	97	0	3	0
Were the study subjects and the setting described in detail?	90	10	0	0
Was the exposure measured in a valid and reliable way?	97	3	0	0
Were objective, standard criteria used for measurement of the condition?	39	29	0	35
Were confounding factors identified?	81	16	3	0
Were strategies to deal with confounding factors stated?	81	16	3	0
Were the outcomes measured in a valid and reliable way?	87	10	0	3
Was appropriate statistical analysis used?	87	13	0	0
Were demographically comparable HIV negative controls included and/or were demographically corrected cognitive scores used?	61	13	26	0

“Yes” means the study fully meets the criteria for this question. “Partly” means the study only partly meets the criteria. “No” means the study does not meet the criteria for this question at all. “NA” means this question is not applicable to this study

In the review context, we defined confounding factors as major neurological or psychiatric conditions. Age-related conditions were not considered under this item as all studies did not assess this question optimally, so this represents an overall limitation for the current HIV and cognitive aging literature at this moment in time

**Table 6:** Rating on quality appraisal among longitudinal studies with the JBI tools

	Yes (%)	Partly (%)	No (%)	NA (%)
Were the two groups similar and recruited from the same population?	83	0	0	17
Were the exposures measured similarly to assign people to both exposed and unexposed groups?	83	0	0	17
Was the exposure measured in a valid and reliable way?	100	0	0	0
Were objective, standard criteria used for measurement of the condition?	17	17	0	67
Were confounding factors identified?	83	17	0	0
Were strategies to deal with confounding factors stated?	83	17	0	0
Were the groups/participants free of the outcome at the start of the study (or at the moment of exposure)?	17	0	0	83
Were the outcomes measured in a valid and reliable way?	100	0	0	0
Was the follow up time reported and sufficient to be long enough for outcomes to occur?	83	0	0	17
Was follow up complete, and if not, were the reasons to loss to follow up described and explored?	50	17	0	33
Were strategies to address incomplete follow up utilized?	33	0	17	50
Was appropriate statistical analysis used?	100	0	0	0
Were demographically comparable HIV negative controls included and/or were demographically corrected cognitive scores used?	33	0	67	0

“Yes” means the study fully meets the criteria for this question. “Partly” means the study only partly meets the criteria. “No” means the study does not meet the criteria for this question at all. “NA” means this question is not applicable to this study

### Narrative synthesis

#### Studies with an HIV- control group and the evidence of premature, accentuated and accelerated neurocognitive aging

Out of 22 studies that included an HIV- sample, 17 studies were cross-sectional and five studies [29, 42–45] were longitudinal. Fifteen cross-sectional studies aimed to find the interaction effect of HIV and age on a continuous measure of neurocognitive test performance whereas two studies [32, 40] examined the interaction effect of HIV and age on both NCI and neurocognitive test performance scores. Among the five longitudinal studies, four studies tested the interaction effect of HIV and age on neurocognitive test performance while one study [42] assessed the interaction effect of HIV and age on incident NCI. In one of the longitudinal studies, the analysis was designed to test the interaction effect of HIV and age only on cross-sectional neurocognitive test performance rather than neurocognitive decline across the study period [43]. Two longitudinal studies [44, 45] examined the interaction effect of HIV and age on both cross-sectional and longitudinal neurocognitive test performance.

Premature neurocognitive aging effect was examined in 17 cross-sectional studies and three longitudinal studies that examined the HIV and age interaction effect on cross-sectional neurocognitive test performance. Of these studies, 9/20 (45%; eight cross-sectional and one longitudinal) showed evidence of premature neurocognitive aging. In all but one study [53], this was attributed to the significantly inferior performance of older HIV + compared to the HIV- participants. In one study [53], the interaction effect came from the superior performance of younger HIV- participants.

Accentuated neurocognitive aging effect was investigated in two cross-sectional studies, which tested the HIV and age interaction effect on the number of tests performed under a defined cut-off score. Neither of these studies found evidence of accentuated neurocognitive aging.

Accelerated neurocognitive aging effect was assessed in four longitudinal studies that analyzed the HIV and age interaction effect on longitudinal neurocognitive test performance as decline in performance across the study period. Accelerated aging was detected in three studies (75%), where neurocognitive decline was significantly greater among older HIV + compared to HIV- individuals [29, 44, 45].

Among 10 studies that did not observe any of the abnormal patterns of neurocognitive aging, the poorest neurocognitive test performance was still observed among older HIV + participants in two of the studies [32, 66].

**Fig. 2:**
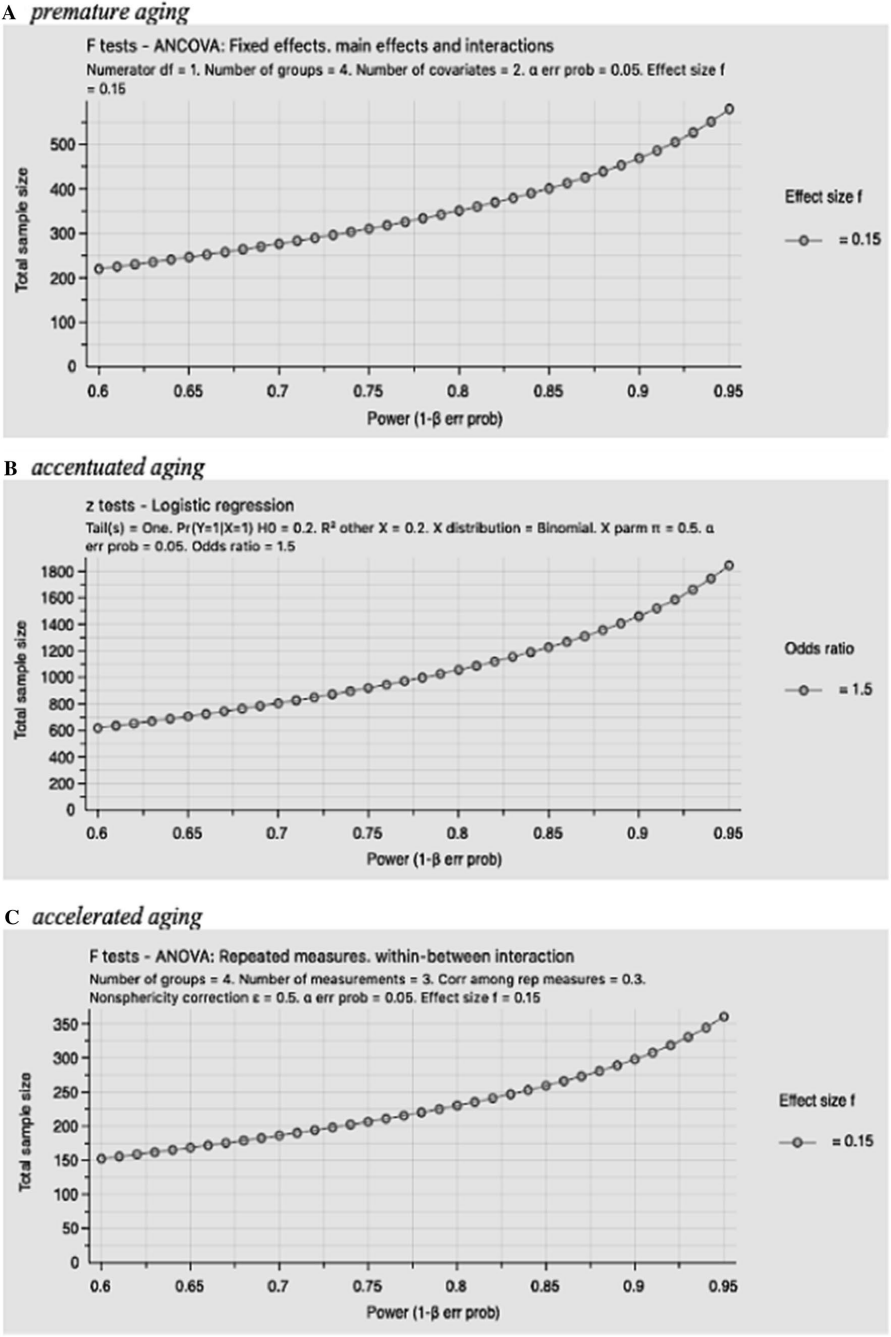
Sample size estimation to detect a small-medium effect size of premature and accentuated aging in a sample statistical model. G*Power 3.1 was used for these calculations. For premature aging, we assume a small-medium effect size, F = 0.15. Four groups are younger HIV-, older HIV-, younger HIV + , and older HIV + . The number of covariates is referred from the median number of covariates among studies that have assessed premature neurocognitive aging. For accentuated aging we assume odds ratio = 1.5 (independent age effect) and 20% of explanatory variance from other covariates. For accelerated aging, we assume the same effect size and the same number of groups as in premature aging with 3 testing, a correlation among repeated measures (r = .3), and a nonsphericity correction at 0.5 representing variations in test retest variance

In evaluating whether study and sample characteristics were related to any of the abnormal neurocognitive aging effects, we found that longitudinally designed studies were more likely to observe premature or accelerated neurocognitive aging effect (80% versus 47%). Sample size also appeared to contribute to the outcomes of interest. Indeed, the three studies [33, 43, 44] that had sample sizes of N > 500, all found evidence for premature or accelerated neurocognitive aging. In studies testing premature aging (N = 20), the median sample size was 189 (IQR: 132.25–304.25); in studies testing accentuated aging (N = 2), the median sample size was 216.5 (IQR:192–241); and in the longitudinal studies assessing accelerated aging (N = 4), the median sample size was 115 (IQR: 62.25–993.5). These figures highlighted that sample sizes in majority of studies are below the required sample size of 350 for premature aging, 1,050 for accentuated aging, and 230 for accelerated aging (see Fig. 2 for detailed sample size computations with G*Power 3.1 [67]) to achieve a conventional power of 80% in order to detect a small-medium effect size of the chronological age effect. Percentage of older people in the total participants also seemed to be associated with whether the hypothesized neurocognitive aging effect was observed or not. Indeed, when ≥ 50% of the sample were over 50 years of age, 67% of studies reported the hypothesized neurocognitive aging effect compared to only 50% of studies where less than 50% of samples were over 50 years. Studies concentrating on NCI rather than on overall neurocognitive test performance were less likely to find the neurocognitive aging effect (i.e., accentuated or accelerated aging). As such, none out of three studies [32, 40, 42] which focused on NCI found abnormal neurocognitive aging.

HIV disease characteristics may also be related to whether premature, accentuated, and/or accelerated neurocognitive aging effects were observed. Indeed, 3/4 studies that recruited only HIV + participants with viral suppression did not find any abnormal neurocognitive aging effect. In studies where standard HIV disease staging was used (WHO or CDC), a higher proportion that reported abnormal neurocognitive aging effects than those that did not (71% versus 33%) included HIV + participants, with at least 50% being diagnosed with a WHO Stage 4 or CDC Stage C. In terms of nadir CD4 + T cell count, a higher percentage of studies that found abnormal neurocognitive aging effects compared to those that did not (56% versus 38%) was composed of HIV + individuals with an average nadir CD4 + T cell count < 200 copies/ml. A greater proportion of studies that showed abnormal patterns of neurocognitive aging (73% vs 50%) also reported a mean/median duration of HIV infection ≥ 12 years among their HIV + participants. Finally, among the seven studies which reported that depressive symptoms were significantly more common among PLHIV than HIV- controls, six studies (86%) observed that abnormal patterns of neurocognitive aging were present.

#### Studies without an HIV- controls group and the magnitude of the age effect on cognition

Among 15 studies that did not include HIV- participants, the presence of an effect of age on neurocognition was observed among the HIV + participants in all the studies. In studies with NCI (N = 7) as the outcome, the odds ratio (OR) of having NCI among older HIV + compared to their younger counterparts varied between 1.18 and 4.8 indicating a higher risk of NCI among older PLHIV compared to the younger people. The effect sizes among the studies which used continuous neurocognitive performance are not reported because most of these studies did not use demographically-corrected cognitive scores.

## Discussion

This is the first systematic review in the area of HIV and neurocognitive aging. This review exhaustively searched four scientific databases to find the relevant literature and determined the current known evidence of premature, accentuated, and accelerated neurocognitive aging among PLHIV and its associated factors by critically and independently appraising the 37 eligible articles. Our review also provides strong evidence of major limitations in the selected studies.

We found that 62% studies met the criteria fully or partly for all the quality assessment items evaluated with the JBI tools. Previous non-systematic reviews have not evaluated study quality with standard quality tools. However, the studies’ heterogeneity in design (cross-sectional/longitudinal), operationalization of age as a predictor, definition of NCI and continuous neuropsychological outcomes (global/domain/focused, various types of tests and various test-score standardization methods), in addition to the statistical and conceptual operationalization of aging effects on neurocognition did not permit for a meta-analysis. This represents the number one limitation across this research literature. One of the most illustrative aspects in the lack of harmonization across this literature is that despite the availability of standard diagnostic criteria for HAND [13, 68], only 22% of studies used these criteria to report HAND prevalence. This is true, despite the majority of studies (68%) using large enough test batteries to compute the neurocognitive severity, part of the criteria.

The second major limitation is that 14/37 studies (38%) included no control samples. It should be clearly stated that while the age effect can be tested on neurocognition in studies without a control group, these studies are fundamentally hindered in their capacity to test premature, accentuated and accelerated aging effects. In other words, these studies offer limited information as to whether PLHIV are at a greater risk of abnormal brain aging and ultimately dementia. In addition, quality appraisal with the JBI tools found that in six of the studies with a control group age was not comparable between HIV + and HIV- groups and demographically-corrected cognitive scores were not used. Six of the studies that did not include a control group also did not use demographically-corrected cognitive scores. To differentiate cognitive aging effects due to HIV from normal aging effect, HIV- controls need to be matched with HIV + participants, at least in age and preferably in other demographic factors as well. If HIV- controls are not age-comparable or if a control group is not included and the question is about whether there is an abnormally large aging effect in PLHIV, then demographically corrected cognitive scores need to be used.

The neurocognitive aging effects in studies that did not include an HIV- control group were consistent but had a wide range. The wide range of effect suggests that the prevalence of HAND and associated HIV disease factors are widely heterogeneous across studies. The higher risk of NCI among older PLHIV compared to younger PLHIV may be attributed to greater exposure to other risk factors of HAND such as a diagnosis with a more severe HIV clinical disease stage, living with HIV without ART for a longer duration, low baseline CD4 count, and exposure to neurotoxic ART drugs [24]. On the other hand, there are likely some embedded survivor effects that have not been taken into account [12].

Previous non-systematic reviews of the HIV and neurocognitive aging literature [22–24] showed that premature and accelerated neurocognitive aging effects were inconsistent, while the accentuated aging was not conceptualized as a separate effect of interest. Using systematic definitions for each abnormal patterns of neurocognitive aging, our systematic review provides a systematic appraisal of this inconsistency. Among the studies that have investigated premature aging (N = 20), 45% reported premature aging. No study found evidence of accentuated aging, though this was only investigated in two studies representing another major limitation of the current HIV neurocognitive aging research. Among the studies that investigated accelerated neurocognitive aging (N = 4), 75% reported accelerated aging. Our systematic review also found that study and sample characteristics were associated with the likelihood of reporting abnormal neurocognitive aging. In the following section, we discuss each of these findings in detail.

This review showed that all three studies (two longitudinal and one cross-sectional) where sample size was greater than 500 reported an abnormal pattern of cognitive aging effect. The lack of large enough sample sizes to detect small-medium effect sizes in most other studies as assessed by our sample size computation could account for inconsistencies in the detection of abnormal patterns of cognitive aging. This represents a major issue especially when we consider that cognitive aging in PLHIV is multifactorial [69]. Besides the effect of chronological age itself, other factors such as HIV characteristics, resilience, lifestyle factors, mental health, age-related comorbidity burden, and aged-related genetic factors need to be individually considered, in addition to their interactions (including complex interaction levels, such as full factorial model and/or polynomials). The current studies typically included 1–3 covariates and thus did not address multi-factorial aging effects. A dataset which would take into account multi-dimensional aging effects would lead to a high-dimensional dataset that typically requires very large sample size whether it is for traditional statistical analyses or data-driven methods (e.g., machine learning) [70]. Reaching such a sample size may only be feasible by prospectively co-enrolling HIV + and HIV- participants in the multitude of existing NeuroHIV studies worldwide. This is not an unrealistic effort, considering it is in progress in dementia research such as International Centenarian Consortium-dementia [71] or neuroimaging research (e.g. Alzheimer’s Disease Neuroimaging Initiative) [72] to cite a few examples. However, data and methods harmonization are a pre-requisite [73].

As already recommended in the standard diagnostic criteria for HAND [13], core minimal data requirements for data pooling should include the coverage of at least five neurocognitive domains, and collection of major confounding conditions such as major neurological and psychiatric disorders, mood disorders, substance/alcohol use disorder. This is not trivial as some of the reviewed studies did not report on HIV characteristics and other major confounders. Standardization in the collection, operationalization and scoring of these data would also be needed, which represents a major effort.

What would constitute an even greater effort in the data pooling is the addition of age-related conditions. In the current review, only a few studies collected information on age-related comorbidities such as CVD (4/37 studies) and hypertension (10/37 studies), which represents one of the most important limitations of the current literature in terms of its immediate clinical relevance. As PLHIV age, we know that comorbidities will play a greater role on neurocognitive decline in addition to chronological age itself [74]. In dementia research, there is increasing recognition that preventable mid-life conditions all represent a cumulative risk factor for dementia [75]. From a clinical perspective, it is imperative to understand the contributions of these comorbidities on NCI among PLHIV for the development of better prevention and clinical management strategies [76], as it may represent one of the best ways to reduce dementia risk in PLHIV. This may also help understanding what the dementia risk may be in PLHIV with low comorbidity burden.

The availability of this kind of data pooling and sharing system could be globally promoted as this is in line with NIH policies and other public research funding agencies around the world [77]. This mechanism would be especially beneficial to smaller studies from resource-limited settings and low-medium income countries with emerging aging HIV populations [78]. In this review, a major limitation is that no study was from low income countries and no research has been conducted on HIV and neurocognitive aging in sub-Saharan Africa [79], where HIV rates are the highest globally [80], where there is an aging HIV population with historical AIDS and rising multi-comorbidities [81, 82], and where HAND prevalence rates range from 16%—80% [83].

In order for this sharing platform to be effective internationally, the NeuroHIV community should agree on a core neuropsychological test battery allowing for the use of HAND diagnostic criteria that can be applied across countries with relative ease. A major challenge with the current diagnostic criteria is the requirement of a functional assessment, and that there is currently not a culturally appropriate functional scale for use in many low-medium income settings. Therefore, an improved algorithm compared to what has been published in 2007 [13] that includes a cross-culturally valid functional assessment would enable more comparable research in the future [14]. Standardization in the collection, operationalization and scoring of these data would also be needed.

The proportion of older persons (over 50 years old) in the total sample may have had some effect on whether abnormal patterns of neurocognitive aging were detected. A higher proportion of studies found the interaction effect of age and HIV on neurocognition when more than 50% of their participants were older PLHIV. The low proportion of older participants may mean low percentage of participants over 60 years old, the age when normal neurocognitive decline starts to increase in the normal population [84]. The lower number of participants over 60 was reported as a limitation in some of the studies in this review that did not find an interaction effect between HIV and age on neurocognition [32, 36, 39, 40]. This limitation across the HIV neurocognitive aging literature further demonstrates that follow-up of the established NeuroHIV cohort studies need long-term support.

The clinical characteristics of HIV + participants had some impact on the finding of premature, accentuated, and accelerated neurocognitive aging effects. Abnormal patterns of neurocognitive aging were observed more commonly in studies where HIV + participants had a higher rate of known risk factors for HAND such as CDC Stage C or WHO Clinical Stage IV and lower baseline CD4 count (< 200). This review also identified the possible “age-duration effect” [19]. Studies which included HIV + participants with longer duration of HIV infection tended to find an interaction effect between HIV and age. This indicates that even between two groups of older HIV + persons with similar age, the group with longer duration of HIV may have lower neurocognitive test performance. Indeed, HIV + persons who have lived with HIV for a longer period of time may have had to experience longer duration of chronic immune activation and inflammation [85]. Future HIV and neurocognitive aging studies should incorporate the duration of HIV infection as a covariate in their analysis in order to be able to differentiate whether abnormal patterns of neurocognitive aging are dependent on either or both chronological age and/or duration of HIV infection.

Studies where HIV + participants had a higher prevalence of depressive symptoms than HIV- participants tended to find abnormal patterns of neurocognitive aging. This is in line with findings that showed that mood disorders may accelerate brain aging [86, 87], and are known independent risk factors for dementia [87]. Because most studies excluded PLHIV with more severe form of psychiatric distress, the neurocognitive aging profile in PLHIV in those with high psychiatric burden remains unknown, and this represents another shortcoming from the current literature. From a clinical perspective, continued investment in bettering the mental health of PLHIV should become a central part of their care as they age.

Our systematic review has the following limitations. We recognize that this review included only articles from peer-reviewed journals which were written in English, and that this may have led to a selection bias. However, NeuroHIV literature has been traditionally published in English, even when from non-English speaking countries, therefore the bias is limited. Furthermore, it is possible that more negative findings exist but were not published. This effect is potentially moderated by the fact that the question of premature, accentuated and accelerated neurocognitive aging among PLHIV is highly debated [22, 88], and thus, that negative findings have been considered as important as positive findings. Our review concentrated on neurocognitive rather than brain aging (e.g., imaging studies) and it is possible that brain changes in some instances precede neurocognitive aging. As some of the studies in this review included only MSM participants (6 studies) and male participants (2 studies), the findings may not fully be relevant to female HIV populations. Since none of studies came from low-income countries, our findings may not be directly applicable to PLHIV from low-income countries. We conceptualized aging as chronological age, however as we have discussed above, aging is a multifactorial process that should ideally be captured multi-dimensionally. Our review shows however, that the current state of the HIV and neurocognitive aging literature would have not permitted the inclusion of other makers of aging.

To conclude, the proportion of PLHIV reaching 60 years of age is ever increasing, an age at which dementia prevalence starts to rise in the general population. Importantly age is the #1 risk factor for dementia. Therefore, even small effects of premature, accentuated or accelerated neurocognitive aging in PLHIV may have major public health implications for dementia risk at the global HIV population level. Evidence for premature neurocognitive aging was inconsistent. Evidence for accelerated neurocognitive aging was consistent but was based on a small number of studies, albeit the largest. Evidence for accentuated neurocognitive aging was rarely investigated and negative when tested. A stronger level of evidence is critically needed to inform clinicians and the HIV care sector about dementia risk among PLHIV to properly facilitate dementia screening, prevention and treatment among aging PLHIV. We propose that to determine accurate estimates of abnormal patterns of neurocognitive aging in PLHIV at the meta-analytical and epidemiological levels, an international effort should be made to collect harmonized longitudinal neurocognitive and other relevant data (including selected key neurological, psychiatric, age-comorbidities and other age markers, that is a high-dimensional dataset) in a very large number. This is needed to enable testing typical small-to medium chronological age effect (d = 0.3) on standard neuropsychological testing [89]. Ideally, this sample would be composed of PLHIV and local age-matched controls that comprise a good representation of low- and middle-income countries and at least 50% with age over 50 + years old.

## Electronic supplementary material

Below is the link to the electronic supplementary material.

Supplementary file1 (DOCX 16 kb)

Supplementary file2 (DOCX 16 kb)

Supplementary file3 (DOCX 36 kb)
