# Supplementary material for: Is There Any Evidence of Premature, Accentuated and Accelerated Aging Effects on Neurocognition in People Living with HIV? A Systematic Review
Source: AIDS Behav. 2020 Oct 6;25(3):917–60. doi: 10.1007/s10461-020-03053-3 (PMC7886778; doi:10.1007/s10461-020-03053-3)
Supplement: Supplementary file 1 — Supplementary file1 (DOCX 16 kb) [file 10461_2020_3053_MOESM1_ESM.docx]

**Search Terms**

| Human Immunodeficiency Virus  HIV  HIV-1  HIV type 1  AIDS | Neurocognitive  Cognitive  Cognition  Neuropsychiatric  Neuropsychological  Neuropsychology  Neuropsychological functions | Disorder  Impairment  Deficit  Disease  Decline  Deterioration  Dementia  HAND  ANI  MND  HAD | Age  Ageing  Old*  Elder* |
| --- | --- | --- | --- |

“OR” was placed between search terms in each column and “AND” was placed between different columns.

**Exemplary search in Embase Database**

(HIV* or Human Immunodeficiency Virus or Acquired Immune Deficiency Syndrome).ti. and (neurocognitive or cognitive or cognition or Neuropsychiatric or Neuropsycholog*).ab. and (Disorder* or Impairment* or deficit* or decline or performance or function* or disease* or deterioration or dementia or defect* or accentuat* or accelerat* or dysfunction* or status* activit* or abilit* or outcome* or dispersion).ti. and (age* or aging or old* or elder*).ti.
