# Supplementary material for: Is There Any Evidence of Premature, Accentuated and Accelerated Aging Effects on Neurocognition in People Living with HIV? A Systematic Review
Source: AIDS Behav. 2020 Oct 6;25(3):917–60. doi: 10.1007/s10461-020-03053-3 (PMC7886778; doi:10.1007/s10461-020-03053-3)
Supplement: Supplementary file 2 — Supplementary file2 (DOCX 16 kb) [file 10461_2020_3053_MOESM2_ESM.docx]

**Data extracted from each study**

- Study number
- Title
- Author
- Year
- Journal
- Country
- Study aims
- Context (inclusion and exclusion criteria, study setting, and any unique characteristics of the study)
- Study design (cross-sectional or longitudinal)
- Sample size
- Age range
- Proportion of elders (≥ 50 years)
- HIV negative control (Yes or NO)
- If no, were neurocognitive scores were demographically corrected with an appropriated normative data?
- Percentage of ART patients
- Percentage of plasma viral suppression or mean/median plasma viral load count
- Percentage of CSF viral suppression or mean/median CSF viral load count
- Percentage by disease stage (AIDS or CDC Stage 3 or WHO Stage IV)
- Mean/median nadir CD4 Count
- Mean/median current CD4 count
- Mean/median duration of HIV infection or serostatus
- Mean/median duration of ART
- Main exposure variables
- Key comorbidities
- Were key comorbidities equally represented among the study samples?
- Neurocognitive domains and tests
- Comprehensiveness of Neuropsychological battery (small/screen; medium (=5 cognitive domains); large)
- How outcome is measured and presented
- Statistical methods
- Effect measure
- Controlled variables
- Prevalence of HAND/NCI
- Results
- Study strengths and limitations
- Summary/Comment
