# Supplementary material for: Is There Any Evidence of Premature, Accentuated and Accelerated Aging Effects on Neurocognition in People Living with HIV? A Systematic Review
Source: AIDS Behav. 2020 Oct 6;25(3):917–60. doi: 10.1007/s10461-020-03053-3 (PMC7886778; doi:10.1007/s10461-020-03053-3)
Supplement: Supplementary file 3 — Supplementary file3 (DOCX 36 kb) [file 10461_2020_3053_MOESM3_ESM.docx]

**Table I: JBI critical appraisal tools for cross-sectional observational studies**

|  | Yes | Partly | No | Not Applicable |
| --- | --- | --- | --- | --- |
| Were the criteria for inclusion in the sample clearly defined? | Clear inclusion and exclusion criteria for study participants were mentioned. | Mentioned. Not clear though. | Inclusion and exclusion criteria were not stated. |  |
| Were the study subjects and the setting described in detail?  Were participants recruited from the community or a HIV clinic or a research project? Were patients from a specific risk group such as MSM or from the general community? Were demographic characteristics of participants reported? | Comprehensive information on the characteristics of study participants and research settings | Information not complete. | No information on this was provided. |  |
| Was the exposure measured in a valid and reliable way? Was chronological age used as a main exposure variable? If age is grouped, is the lower cut-off for older age group 50 years at a minimal? | If age is grouped, the lower cut-off for older age group is 50 years at minimum. | The lower cut-off for older age group is between 40-50 years. | The lower cut-off for older age group is <50 years. |  |
| Were objective, standard criteria used for measurement of the condition?  If neurocognitive impairment was identified, was American Academy of Neurology (AAN) criteria or Frascati criteria or any objective criteria used for diagnosis? | AAN or Frascati criteria was used, and correctly applied. | Their own objective criteria was used. | No objective criteria was pursued. | The main outcome was not neurocognitive impairment. |
| Were confounding factors identified?  Were possible confounders (e.g., psychiatric disorders) and contributing conditions (e.g., previous history of substance use) identified? | At least, major confounders such as major neurological disorders, major psychiatric disorders, history of traumatic brain injury with loss of consciousness more than 30 minutes, CNS opportunistic infections, current substance abuse, and severe education/developmental disability were identified. | Not all major confounders were identified. | No confounders were identified. |  |
| Were strategies to deal with confounding factors stated? Were possible confounders (e.g., psychiatric disorders) and contributing conditions (e.g., previous history of substance use) controlled for? | Attempts to control for at least major confounders. | Not all major confounders were dealt with. | No attempts to control for confounders at all. |  |
| Were the outcomes measured in a valid and reliable way?  What neuropsychological tools were used? | Proper neuropsychological batteries were used. | At least, a screening tool such as International HIV Dementia Scale (IHDS) was used. | No objective measures were used. | Selective tools were used to test on a specific neuropsychological domain. |
| Was appropriate statistical analysis used? Were appropriate statistical methods used to test the hypothesis and answer the research question? Were analysis intended to identify and control for confounding factors? | Statistical analyses were strong enough to answer the research questions, and to identify and control for confounders. | Analyses were appropriate to test the hypothesis but not to identify and control possible confounders. | Analyses were not relevant to answer the study objectives. |  |
| Were demographically comparable HIV negative controls included and/or were demographically corrected cognitive scores used? | Included demographically comparable HIV negative controls and/or used demographically corrected cognitive scores | Included controls which are comparable with HIV positive participants in terms of age, but not in other demographic factors, and did not use demographically corrected cognitive scores. | Did not include age-matched HIV negative controls or did not use demographically corrected cognitive scores. |  |

**Table II: JBI critical appraisal tools for longitudinal observational studies**

|  | Yes | Partly | No | Not Applicable | |
| --- | --- | --- | --- | --- | --- |
| Were the two groups similar and recruited from the same population?  Were similar inclusion and exclusion criteria applied to both HIV positive and control groups? | Similar inclusion and exclusion criteria were applied to both HIV positive and control groups |  | Different inclusion and exclusion criteria were applied to HIV positive and control groups | No control group | |
| Were the exposures measured similarly to assign people to both exposed and unexposed groups?  If HIV negative controls were included, was similar age grouping assigned to both HIV positive and control groups? | Similar age grouping was assigned to both HIV positive and control groups. |  | Different age grouping was assigned to HIV positive and control groups. | No control group | |
| Was the exposure measured in a valid and reliable way? Was chronological age used as a main exposure variable? If age is grouped, is the lower cut-off for older age group 50 years at a minimal? | If age is grouped, the lower cut-off for older age group is 50 years at a minimal. | The lower cut-off for older age group is between 40-50 years. | The lower cut-off for older age group is <50 years. | |  |
| Were objective, standard criteria used for measurement of the condition?  If neurocognitive impairment was identified, was American Academy of Neurology (AAN) criteria or Frascati criteria or any objective criteria used for diagnosis? | AAN or Frascati criteria was used, and correctly applied. | Their own objective criteria was used. | No objective criteria was used. | The main outcome was not neurocognitive impairment. | |
| Were confounding factors identified?  Were possible confounders (e.g., psychiatric disorders) and contributing conditions (e.g., previous history of substance use) identified? | At least, major confounders such as major neurological disorders, major psychiatric disorders, history of traumatic brain injury with loss of consciousness more than 30 minutes, CNS opportunistic infections, current substance abuse, and developmental disability were identified. | Not all major confounders were identified. | No confounders were identified. |  | |
| Were strategies to deal with confounding factors stated?  Were possible confounders (e.g., psychiatric disorders) and contributing conditions (e.g., previous history of substance use) controlled for? | Attempts to control for at least major confounders. | Not all major confounders were dealt with. | No attempts to control for confounders at all. |  | |
| Were the groups/participants free of the outcome at the start of the study (or at the moment of exposure)?  If the outcome is neurocognitive impairment, were participants free from this at the start of the study? | All the participants were free from neurocognitive impairment. |  | Not all participants were free from neurocognitive impairment. | For studies which compared only the neuropsychological function or performance. | |
| Were the outcomes measured in a valid and reliable way?  What neuropsychological tools were used? | Proper neuropsychological batteries were used. | At least, a screening tool such as IHDS was used. | No objective measures were used. | Specific tools were used to test on a specific neuropsychological domain. | |
| Was the follow up time reported and sufficient to be long enough for outcomes to occur? | ≥ 1 year | 6-12 months | < 6 months |  | |
| Was follow up complete, and if not, were the reasons to loss to follow up described and explored? | At least 80% participants attended the follow-up screening. | More than 20% of patients did not complete follow-up, but the reasons were detailed. | More than 20% of patients did not complete follow-up, and the reasons were not detailed. |  | |
| Were strategies to address incomplete follow up utilized? Were efforts made to obtain the outcome of participants who dropped out and did analyses try to account for shorter time follow-up among these participants? | The study attempted to obtain outcome from those who did not finish follow-up and analyses tried to account for shorter time follow-up among these participants. | The study attempted to obtain outcome from those who did not finish follow-up, but analyses were not adjusted to account for shorter time follow-up among these participants. | The study did not attempt to obtain outcome from those who did not finish follow-up, and analyses were not adjusted to account for shorter time follow-up among these participants. | All completed the follow-up | |
| Was appropriate statistical analysis used? Were appropriate statistical methods used to test the hypothesis and answer the research question? Were analyses intended to identify and control for confounding factors? | Statistical analyses were strong enough to answer the research questions and to identify and control for confounders | Analyses were appropriate to test the hypothesis but not to identify and control possible confounders. | Analyses were not relevant to answer the study objectives. |  | |
| Were demographically comparable HIV negative controls included and/or were demographically corrected cognitive scores used? | Included demographically comparable HIV negative controls and/or used demographically corrected cognitive scores. | Included controls which are comparable with HIV positive participants in terms of age, but not in other demographic factors, and did not use demographically corrected cognitive scores. | Did not include age-matched HIV negative controls or did not use demographically corrected cognitive scores. |  | |

**Table III: Quality appraisal findings for cross-sectional studies**

|  | **Were the criteria for inclusion in the sample clearly defined?** | **Were the study subjects and the setting described in detail?** | **Was the exposure measured in a valid and reliable way?** | ***Were objective, standard criteria used for measurement of the condition?*** | **Were confounding factors identified?** | **Were strategies to deal with confounding factors stated?** | **Were the outcomes measured in a valid and reliable way?** | **Was appropriate statistical analysis used?** | **Were demographically comparable HIV negative controls included and/or were demographically corrected cognitive scores used?** |
| --- | --- | --- | --- | --- | --- | --- | --- | --- | --- |
| **Sheppard, et al., 2017** | Yes | Yes | Yes | NA | Yes | Yes | Yes | Yes | Yes |
| **Kupprat, et al., 2015** | Yes | Yes | Yes | NA | Partly | Partly | Yes | Partly | No |
| **Pinheiro, et al., 2016** | Yes | Yes | Yes | Partly | Partly | Partly | Partly | Yes | No |
| **Kinai, et al., 2017** | Yes | Yes | Yes | Yes | Yes | Yes | Yes | Yes | Yes |
| **Vance et al. 2011** | Yes | Yes | Yes | NA | Yes | Yes | Yes | Yes | No |
| **Ludicello, et al., 2012** | Yes | Yes | Yes | Yes | Yes | Yes | Yes | Yes | Yes |
| **Ciccarelli et al. 2012** | Yes | Yes | Yes | Partly | Yes | Yes | Yes | Yes | Yes |
| **Gawron, et al., 2018** | Yes | Yes | Yes | Yes | Yes | Yes | Yes | Yes | Yes |
| **Cherner, et al., 2004** | Yes | Yes | Yes | *Yes* | Yes | Yes | Yes | Yes | Yes |
| **Cohen, et al., 2019** | Yes | Yes | Yes | NA | Yes | Yes | Yes | Yes | Yes |
| **Valcour, et al., 2004a** | Yes | Yes | Yes | Yes | Yes | Yes | Yes | Yes | Yes |
| **Cysique et al. 2011b** | Yes | Yes | Yes | Yes | Yes | Yes | Yes | Yes | Yes |
| **Pluta, et al., 2019** | Yes | Yes | Yes | Yes | Yes | Yes | Yes | Yes | Yes |
| **Coban, et al., 2017** | Yes | Yes | Yes | Partly | Partly | Partly | Yes | Yes | Yes |
| **Ding, et al., 2017** | Yes | Yes | Yes | Partly | Partly | Partly | Partly | Yes | Yes |
| **Towgood, et al., 2012** | Yes | Yes | Yes | NA | Yes | Yes | Yes | Partly | Yes |
| **Sacktor, et al., 2007** | Yes | Yes | Yes | Yes | Yes | Yes | Yes | Yes | Yes |
| **Tan, et al., 2013** | Yes | Yes | Yes | Yes | Yes | Yes | Yes | Yes | Yes |
| **Sandkovsky, et al., 2013** | Yes | Yes | Yes | NA | Yes | Yes | Yes | Partly | Yes |
| **Valcour, et al., 2011** | Yes | Yes | Yes | Partly | Yes | Yes | Yes | Yes | Yes |
| **Van Dyk, et al., 2015** | Yes | Yes | Yes | NA | Yes | Yes | NA | Yes | No |
| **Avci, et al., 2016** | Yes | Yes | Yes | NA | Yes | Yes | Yes | Yes | Partly |
| **Vance, Fazeli and Gakumo 2013** | Yes | Yes | Yes | NA | Yes | Yes | Yes | Yes | Partly |
| **Kissel, Pukay-Martin and Bornstein 2005** | Yes | Partly | Partly | *Partly* | Yes | Yes | Yes | Yes | Partly |
| **Panos, et al., 2013** | Yes | Yes | Yes | Yes | Yes | Yes | Yes | Yes | No |
| **Foley, et al., 2010** | Yes | Yes | Yes | NA | Yes | Yes | Yes | Yes | No |
| **Morgan, et al., 2011** | Yes | Yes | Yes | NA | Yes | Yes | Yes | Yes | Yes |
| **Scott, et al., 2011** | Yes | Yes | Yes | Yes | Yes | Yes | Yes | Yes | Partly |
| **Wilkie, et al., 2003** | No | Yes | Yes | Partly | Partly | Partly | Yes | Yes | Yes |
| **Kim et al. 2008** | Yes | Partly | Yes | NA | No | No | Yes | Partly | No |
| **Xiao, et al., 2019** | Yes | Partly | Yes | *Partly* | Yes | Yes | Partly | Yes | No |

**Table IV: Quality appraisal findings for longitudinal studies**

|  | **Sheppard, et al., 2015** | **Goodkin, et al., 2017** | **Haynes, et al., 2018** | **Sacktor, et al., 2010** | **Seider, et al., 2014** | **Larussa, et al., 2006** |
| --- | --- | --- | --- | --- | --- | --- |
| **Were the two groups similar and recruited from the same population?** | Yes | Yes | Yes | Yes | Yes | NA |
| **Were the exposures measured similarly to assign people to both exposed and unexposed groups?** | Yes | Yes | Yes | Yes | Yes | NA |
| **Was the exposure measured in a valid and reliable way?** | Yes | Yes | Yes | Yes | Yes | Yes |
| **Were objective, standard criteria used for measurement of the condition?** | Yes | NA | NA | NA | NA | Yes |
| **Were confounding factors identified?** | Yes | Yes | Yes | Yes | Yes | Partly |
| **Were strategies to deal with confounding factors stated?** | Yes | Yes | Yes | Yes | Yes | Partly |
| **Were the groups/participants free of the outcome at the start of the study (or at the moment of exposure)?** | Yes | NA | NA | NA | NA | NA |
| **Were the outcomes measured in a valid and reliable way?** | Yes | Yes | Yes | Yes | Yes | Yes |
| **Was the follow up time reported and sufficient to be long enough for outcomes to occur?** | Yes | NA | Yes | Yes | Yes | Yes |
| **Was follow up complete, and if not, were the reasons to loss to follow up described and explored?** | Partly | NA | Yes | NA | Yes | Yes |
| **Were strategies to address incomplete follow up utilized?** | No | Yes | NA | NA | Yes | NA |
| **Was appropriate statistical analysis used?** | Yes | Yes | Yes | Yes | Yes | Yes |
| **Were demographically comparable HIV negative controls included and/or were demographically corrected cognitive scores used?** | Yes | No | No | No | No | Yes |
